# Non-medical practitioners in the staffing of emergency departments and urgent treatment centres in England: a mixed qualitative methods study of policy implementation

**DOI:** 10.1186/s12913-023-10220-4

**Published:** 2023-11-08

**Authors:** Vari M. Drennan, Mary Halter, Francesca Taylor, Jonathan Gabe, Heather Jarman

**Affiliations:** 1 Centre for Applied Health and Social Care Research, Kingston University, Kingston Upon Thames, UK; 2 Royal Holloway, University of London, Egham, UK; 3 St George’s University Hospitals NHS Foundation Trust, London, UK

**Keywords:** Emergency service, Hospital, Workforce, Physician associates, Nurse practitioners, Policy, England

## Abstract

**Background:**

Patient demand, internationally, on emergency departments and urgent care treatment centres has grown. Shortages of staff, particularly of emergency medicine doctors, have compounded problems. Some countries are pursuing solutions of including non-medical practitioners e.g., nurse practitioners and physician associates/assistants in their emergency department workforces. This study investigated at the macro and meso level of the health system in England: what the rationale was and the factors influencing the current and future employment, or otherwise, of non-medical practitioners in emergency departments and urgent treatment centres.

**Methods:**

Mixed qualitative methods in the interpretative tradition were employed. We undertook, in 2021–2022, a documentary analysis of national, regional and subregional policy (2017–2021), followed by semi-structured interviews of a purposive sample (*n* = 18) of stakeholders from national, regional and subregional levels. The data were thematically analysed and then synthesised.

**Results:**

There was general national policy support for increasing the presence of non-medical practitioners as part of the solution to shortages of emergency medicine doctors. However, evidence of policy support dissipated at regional and subregional levels. There were no published numbers for non-medical practitioners in emergency departments, but stakeholders suggested they were relatively small in number, unevenly distributed and faced uncertain growth. While the experience of the COVID-19 pandemic and its aftermath were said to have made senior decision makers more receptive to workforce innovation, many factors contributed to the uncertain growth. These factors included: limited evidence on the relative advantage of including non-medical practitioners; variation in the models of service being pursued to address patient demand on emergency departments and the place of non-medical practitioners within them; the lack of a national workforce plan with clear directives; and the variation in training for non-medical practitioner roles, combined with the lack of regulation of that level of practice.

**Conclusions:**

We identified many features of a system ready to introduce non-medical practitioners in emergency departments and urgent treatment centres but there were uncertainties and the potential for conflict with other professional groups. One area of uncertainty was evidence of relative advantage in including non-medical practitioners in staffing. This requires urgent attention to inform decision making for short- and long-term workforce planning. Further investigation is required to consider whether these findings are generalisable to other specialties, and to similar health systems in other countries.

**Supplementary Information:**

The online version contains supplementary material available at 10.1186/s12913-023-10220-4.

## Background

Health care systems internationally have reported rising patient numbers attending emergency departments and urgent care treatment centres (EDs/UTCs) before and since the first wave of the COVID-19 pandemic [1]. Shortages of staff, particularly of doctors trained in emergency medicine have compounded stresses on these services in many countries, including the United Kingdom (UK), [2]. This combination of issues has resulted in negative patient and staff experiences [3]. One response has been the advocacy by the World Health Organisation (WHO) for new types of clinically trained health professionals, such as nurse practitioners (NPs) and physician associates/assistants (PAs) to undertake some of the work of doctors [4]. These new types of clinical health professionals are educated to undertake medical histories, clinical assessments, order diagnostic tests, make diagnoses and commence treatment for any presenting patients as agreed with medical clinicians and/or employers [4]. These new types of professionals are clinical decision makers with a broader scope of practice than health professionals who follow specific extended practice clinical protocols for specified groups of patients [5]. There is no agreement internationally on collective nomenclature for these types of professionals and we use the term non-medical practitioner (NMP), [6] in this paper. A recent scoping review identified published accounts of the employment of NMPs in ED/UTCs in 12 countries [7]. Pilot or demonstration projects only were identified from Denmark [8], the Netherlands [9], New Zealand [10, 11], Norway [12], Saudi Arabia [13] and Uganda [14]. Reports from Australia, Canada, England, Ireland, Scotland, and the United States (USA) detailed more widespread but far from universal employment of NMPs in EDs/UTCs [15–20]. While there were accounts reporting on the facilitators and challenges in single organisation introduction of an or more than one NMP into an ED/UTCs [21–26], there have been none that investigated explanations for the variation in the extent of employment of NMPs in EDs/UTCs from across a health system. The National Health Service in England has had a national policy of support for NMPs in ED/UTCs and offered an opportunity to investigate across a health system. This paper reports on a study investigating the factors influencing the employment or otherwise of NMPs in EDs/UTCs in one health system, the National Health Service (NHS) in England, from the macro and meso level perspective.

### The context for NHS England ED/UTC workforces

The NHS is a universal tax funded health care system, free at the point of care delivery, and provided by individually governed NHS organisations and other types of not for profit and for-profit providers [27]. The NHS is nationally led by an arms -length body called NHS England which receives its mandate and funding from the government Department of Health & Social Care [27]. NHS England sets out the policies and mechanisms for planning and funding clinical care as well as health workforce planning and training [27].

In 2021–2 there were 24 million attendances at 151 NHS EDs/UTCs [28]. In the same year NHS organisations employed 8,155 full time equivalent (FTE) doctors (5,996 of whom were in training positions), 16,790 FTE nurses and 14,200 FTE other types of care providers (unspecified) in EDs/UTCs [28]. There has been a twenty-year history of developing extended scope of practice roles (particularly for nurses and paramedics) in EDs/UTCs in England [29] to address increased patient demand and medical staffing problems [30]. A few individual NHS hospitals developed and employed NMPs [31]. In 2017 the Royal College of Emergency Medicine (RCEM), with the support of the Royal College of Nursing and College of Paramedics, published a curriculum and credentialling process for emergency care advanced clinical practice (EC-ACPs) [17]. In the same year, NHS England published a policy statement promoting increased numbers of NMPs to be employed in ED/UTCs and invested funding in training more ED/UTC NMPs [32]. Against this national policy support for ED/UTC NMPs, this study investigated at the macro and meso level of the health system: what the rationale was for including NMPs in the ED/UTC workforce; and what factors were influencing the current and future employment, or otherwise, of NMPs in the ED/UTC workforce.

As a study of the influences of the implementation of workforce innovation policy we framed our investigation through Greenhalgh et al.’s theory of the diffusion of innovation in health services [33]. This theory argues for a complex interplay between the nature of the innovation, the system readiness for the innovation, the wider socio-economic context, and the resource system.

## Methods

The mixed qualitative methods design drew on the interpretative tradition, recognising multiple perceptions occurring within specific socio-cultural and historical contexts [34]. In order to explore the research questions at both the macro and meso levels of the health system we undertook two sequential investigations followed by an integration of data phase. We first conducted a documentary analysis of policy, followed by semi-structured interviews of a purposive sample of key stakeholders at national and regional levels. We then integrated the data through a narrative synthesis against the research questions, which is reported in the discussion section of this paper.

Data were collected for the documentary policy analysis [35] by searching the websites of public organisations at the macro and meso level of the NHS in England. We searched only those organisations with authority to direct or influence the use of public NHS funds used for emergency and urgent care services and its workforce. At the macro level of the health system these organisations included: the Department of Health & Social Care, the Parliamentary Health Select Committee, the Care Quality Commission (responsible for inspection of services), NHS England (including the newly merged NHS Improvement organisation), NHS major trauma networks and Health Education England (HEE, then responsible for workforce planning and funding training). At the meso level of the health system we searched websites of regional offices of NHS England and HEE, and of local level organisations and networks involved in planning and commissioning of NHS services (at the time of the study these included Sustainability and Transformation Partnerships, Integrated Care Systems, Clinical Commissioning Groups, Integrated Care Boards). We searched for documents published between January 2017 (the year of the NHS England stated policy support for NMPs in EDs/UTCs) and May 2021 (Table 1).

**Table 1:** Policy document sources

	**Document source**	**Search term and/or document type**	**Number of publicly available documents found in scope**	**Number referring to NMPs in ED/UTC**
**Macro level**	GOV.UK Department of Health & Social Care	Urgent and emergency care /services NHS England Mandate NHS England Performance Assessment NHS Workforce HEE Mandate HEE Performance Assessment	27	5
	Parliament Health Select Committee	Urgent and emergency care/ services	0	0
	NHS England /NHS Improvement	Urgent and emergency care /services, strategic plans, annual report, annual business plan, workforce strategy, annual NHS operational planning and contracting guidance urgent and emergency care	25	9
	Health Education England (HEE)	Urgent and emergency care /service, strategic framework & plans, annual report, annual business plan, workforce strategy for urgent and emergency care advanced practice	9	2
	Care Quality Commission	Reports on emergency and urgent care service	2	2
**Meso level**	NHS England/NHS Improvement Regions (*n* = 7)	Urgent and emergency care /services /work programme, strategic plans, annual report, annual business plan, workforce strategy	0	0
	HEE Regions (*n* = 7)	Urgent and emergency care /services /work programme, strategic plans, annual report, annual business plan, workforce strategy	1	0
	Sustainability and Transformation Partnerships and/or, Integrated Care Systems (STPs/ICS, *n* = 42)	Urgent and emergency care /services /work programme, strategic plans, annual report, annual business plan, workforce strategy, local workforce action boards plans and reports	32	1
	Clinical Commissioning Groups (moving to be integrated care boards ( *N* = 42 of 106, sampled of first one in each STP/ICS April 2021)	Urgent and emergency care /services /work programme, strategic plans, annual report, annual business plan, workforce strategy	12	0
	Major Trauma Networks (*n* = 17, in 2017)	Strategic plans, annual report, annual business plan, workforce strategy	0	0

Policy statements, strategies, plans, directives, guidance, reports, reviews, and evaluations of NMP implementation were included and illustrative case studies or opinions without policy directives or guidance were excluded. The websites of professional bodies (RCEM, Royal College of Nursing, Royal College of Pharmacists, College of Paramedics, and the Chartered Society of Physiotherapists) were also searched for any references to relevant public organisation documents or co-authored documents not identified through other searches. Retrieved documents were logged in an electronic spreadsheet and stored electronically. Documents were word searched using the ‘find’ function for the words: “emergency care”, “urgent care” (context and population of interest), “practitioner”, “associate, “advanced”, “clinical pharmacist” (population of interest), and “skill-mix” (concept of interest). On finding the words of interest, the surrounding paragraphs were read and text relevant to the research questions (e.g., employment, intentions to develop or employ NMPs, related rationales) identified. These data were extracted to a spreadsheet. Inductive and deductive analysis was undertaken [36] and iterated and interpreted though discussion in the research team and at consultative events with patients, non-medical practitioners, clinicians, and managers.

Qualitative semi-structured interviews were conducted with a purposive sample of 18 senior NHS clinicians, managers, commissioners and lay representatives from organisations operating at macro and meso levels: national, regional NHS (including arms-length bodies), and sub-regional NHS organisations and professional organisations between August 2021 and April 2022 (Table 2). Lay representatives were included as they participated in macro and meso level policy influencing and policy making committees.

**Table 2:** Participants in interviews

System level of stakeholders’ role	Number invited	Declined/non- response to second invitation	Agreed	Interviewed
National (macro)	35	26	10	9
Regional and subregional (meso)	29	18	10	9
**Total**	**64**	**44**	**20**	**18**

Potential participants were identified through the website searching for the documentary analysis and invited by publicly available email addresses. A topic guide was constructed from the literature and policy analysis and refined in discussion with the study advisory panels which included patients and NMPs. The topic guide (supplementary file 1) focused on questions about the influences/rationale experienced in decisions to employ/train NMPs, expected benefits and risks, and outcomes regarding use of NMPs in EDs/UTCs and the wider system and any views on the mix of staff and skills in EDs/UTCs. Interviews were conducted by video call, recorded with permission, transcribed, anonymised and the recording deleted. Interviewers (VMD, FT, MH) employed techniques of checking understanding, interpretation and summarising during the interview with participants [37]. Interviews lasted from between 30 and 50 min. Interviewers were all female health services researchers, one with a clinical managerial background, and all with prior experience of research on the topic of non-medical practitioners. Reflexive notes were made immediately after interviews and open discussions held in the team to ensure both consistency of approach and iterative questioning. Transcripts were thematically analysed that allowed for deductive (theory framed) and inductive (data driven) approach [36]. Two members of the team (VMD, FT) initially read and developed a coding index, subsequently iterated and refined through discussion with the wider team.

The interview element of the study was reviewed and approved by the NHS North East—Tyne & Wear South Research Ethics Committee (REC number 21/NE/0071).

## Findings

We report the findings from the documentary policy analysis first and then the interviews. The data are then integrated in the discussion section.

### The documentary policy analysis

At the national macro level of the health system, we identified sixty-three documents in scope published by the Department of Health and Social Care, its arm’s length bodies and NHS England, 25 referred to NMPs and of these 14 referred to NMPs specifically in ED/UTCs. The 14 documents reported the existence of NMPs in some ED/UTC services and national level general support for growth in their numbers in response to stated concerns about shortages of emergency medicine doctors, increased patient demand and decreased quality of emergency services.

“*Professional groups such as advanced clinical practitioners, pharmacist clinicians and physician associates are also being developed and supported to take on collaborative, frontline clinical roles in EDs under the supervision and mentorship of consultants in emergency medicine. These groups form an important part of today’s emergency care workforce, giving it greater resilience and sustainability*” p9 [32]

Aside from the specific allocation in 2017 of national funds to train 42 existing health professionals in 14 NHS organisations to be advanced clinical practitioners (ACPs) in the ED [32], the national statements were of general support for NMPs rather than specific instructions for employment. In the response to a parliamentary select committee recommendation that efforts to improve staffing in EDs be redoubled, the government listed actions on NMPs including the opportunities for ACP training fellowships but no directives to the NHS on their employment [38].

At the meso level of the health care system we noted that the regional offices of NHS England, NHS Improvement, HEE, and the regional major trauma networks had very limited web presence with few publicly available documents. One in-scope document was found but made no reference to NMPs in EDs/UTCs. At the sub regional level, in scope documents were found published by 32 integrated care systems; of which 15 referred to developing NMP posts in support of the service transformation and primary care objectives of the 2019 NHS Long Term Plan [39]. Of these 15 policy statements on NMPs, two specifically referred to NMP development in urgent care settings and one to the consideration of ACP roles in emergency departments but without explicit rationale or actions, as in this exemplar:

*“There are a number of new roles we need to consider in our long-term strategic workforce planning at both a local and whole system level. These include: ………. advanced clinical practice roles (especially A & E, cancer, elective specialities).” Hampshire and Isle of Wight Integrated Care System Strategic Delivery Plan 2019-2024. working draft version 6 last edited 17th February 2020 p 10 *[40]*.*

At the sub-ICS level (clinical commissioning groups transferring to integrated care boards and integrated care partnerships during the period of the study), 12 in scope documents were retrieved. While seven referred to plans for improving urgent and emergency services, none referred to NMPs as part of the solution to identified performance problems.

In summary while there was macro, government level general support for NMPs in EDS/UTCs as a policy solution to shortages of emergency medicine doctors, there were no direct implementation instructions to NHS organisations and at the meso-level of the system we found little policy attention to developing or employing NMPs in EDs/UTCs.

### The stakeholder interviews

We now report on themes identified against our research question from the stakeholder interviews. First, we note how the interviewees contextualised their responses through the experience and impact of the COVID-19 pandemic before discussing views as to rationales for NMPs, factors supporting and inhibiting the development of roles and NMP employment in EDs/UTCs.

#### The COVID-19 pandemic context

All the interviewees commented on the experience of providing health care and health professional education through the COVID-19 pandemic as creating a changed context in comparison to pre-2020. The participants reported the increased demands on services and on an already overstretched workforce. Some participants noted the pandemic period had increased patient and public familiarity with different forms of health care practices and different types of staffing. The pandemic was described as having stimulated rapid change in working practices, deployment / utilisation of different staff groups as well as collaborative ways of meeting challenges.

*“If COVID has taught us anything, it's that where we show strength is when we're working together. Any schemes, any new risks we've taken, any different services we've done, have been in partnership with other professions or other stakeholders. Why would that change when we come out of COVID?”* Participant 18 regional, clinician, manager

These changes were observed to have subsequently created the opportunity to re-think the types of staffing needed in a service, as in this exemplar:

*“We suddenly have to provide COVID care, and we didn't have a workforce to provide that care. We've had to pull it from other bits of the workforce. In doing that, we've realised, I think, where the gaps are across the workforce as a whole. For example, if you pull three junior doctors from an area of medicine to go and work on a COVID ward, …..Is it more junior doctors who need to fill that gap or should you be asking a different question?”* Participant 17 regional, clinician

However, a different view was offered by one participant who argued that the ongoing legacy problems of the COVID-19 pandemic meant commissioners were now paying more attention to the wider system of primary care in a local geography to address the increased problems in emergency services, rather than being interested in the internal provider issues of workforce,

#### Rationale for the development and employment of NMPs in EDs/UTCs

For those participants able to comment on reasons why NMPs were being trained and employed, they reported the long-term medical shortages in emergency medicine as the most significant issue for which NMP posts were seen as one part of the solution.

*“It's* [employing NMPs] *just a new way to bring capacity into the system, and we desperately need it.”* Participant 6, national, policy

“*Unless they're going to suddenly train a whole load of medical students to become doctors, but that's going to take five years, ten years, even if the numbers went up. You've got to think about it differently. You've got to stop thinking hierarchy and think about, if you're a patient and you're ill, who is the best person to treat you, and who can you get to see the patient, and who is available to see the patient?*’’ Participant 10, regional , sub regional , clinician.

Some participants suggested that another driver was the need to provide a greater proportion of permanent members in ED staffing, with concomitant benefits to patient and staff experience as in this exemplar.

*"I think a really important thing now is about the continuity of care, so the junior doctors move round all the time, don't they? They do, like, rotations whereas if you have ACPs or PAs in a department they would be quite long-term….you would get a kind of stability within a department”. Participant* 11, national, layperson

It was also suggested by some participants that NMPs, as part of a stable ED/UTC workforce, contributed to and improved the training of other professionals including doctors. Another rationale linked to workforce stability was reported to be the need to retain experienced emergency service nurses. NMP roles were considered to offer an attractive clinical career pathway and some examples were given of very high application levels for NMP training posts in emergency and urgent care services.

“[NMPs] *are a focus for the nurse career progression and other colleague progression as well…… it's better to retain and give our nurses something to work towards*.” Participant 1, regional, clinician, manager

There was a consensus that NMPs in EDs/UTCs were a relatively small group but were thought to be growing in numbers. Although no one was able to offer exact numbers or specify type of professional background, most participants considered that nurses were in the majority in NMP roles.

“*I sit on a multi-professional rota in practice. In that role, it can't be a registrar. It has to be a general practitioner or an advanced practitioner. In reality, it's still mainly nurses, but could be a paramedic, it could be a pharmacist, or it could be a physio”.* Participant 16, national, clinician

There was also agreement that NMPs were not uniformly employed across the regions of England. One explanation was that emergency medicine workforce problems had not been experienced to the same degree in different regions.

*“It's very patchy across the UK, never mind getting into the devolved nations, how such roles have been adapted, supported, and implemented……As I say, the* [name of region] *is terribly late to the party, for the reason that it hasn't had medical workforce issues, historically.”* Participant 20, national, regional, clinician

#### Factors supporting the NMP employment and development

As reported above the context of the COVID-19 pandemic and its legacy (i.e., increased patient demand in EDs/UTCs and workforce shortages), were seen as very influential in supporting NMP employment. Beyond the pandemic impact, the participants described evidence of the value of NMPs to services, familiarity with NMPs and national and regional policy on NMPs as factors supporting NMP employment and development. The three types of factors were often reported to be intertwined.

#### Evidence of NMP value to the service

Some participants considered that a supporting factor was evidence of benefit to the service. It was commented on that this often took time as it required NMPs to have completed their training programmes, and induction periods in their ED/UTC posts.

*“It wasn't until they were allowed to kind of go through the training, flourish, get to the other end, that they* [medical clinicians] *then began to really see the value, and the ACPs weren't just doing what the consultants wanted them to be able to do. For example, I don't know, manage a trauma safely, be able to request specific imaging and be able to interpret that quickly and efficiently, but actually they then saw them in the light that they [the ACPs] were then beginning to educate their medical trainees; that they were developing, they were looking at ways that service could be improved”* Participant 8, regional, clinician, commissioner

From the patient’s perspective, NMPs were seen to add value by reducing ED/UTC waiting times, making visits more efficient and positive:

*“We know that the public angle is that the demands on urgent care and accident and emergency departments is huge. People are waiting for a very long time, so that makes them very anxious. I think they would be much less anxious seeing a senior practitioner from any type of background than waiting for hours not seeing somebody.”* Participant 5, national, layperson

While some participants speculated that a factor positively influencing the employment of NMPs would be that they were cheaper than doctors, participants with more direct knowledge were able state that NMPs were less costly than locum doctors but not middle grade doctors:

*“ACPs are not cheaper than middle-grade* [doctor] *counterparts; that's not true. In fact, ACPs are ever so slightly, for hour-for-hour of direct patient care, slightly more expensive. However, when you compare that to a locum, which is your only real other alternative to that* [middle grade doctor] *workforce, [ACPs] are considerably less financially burdensome”*. Participant 1, regional, clinician, manager

Many participants viewed the history of incremental development of NMP types of posts, with meso-level system support over many years, as increasing the familiarity of clinicians and managers as particularly helpful.

*“So, the development started* [in the NHS region] *back in 2005, and that was funded by the strategic health authorities at the time, and then it became NHS* [name of region]”. Participant *.*8 regional, clinician, commissioner

Participants reported that NMPs were more widely accepted. The nearly twenty-years of incremental development of NMP posts, combined with recognition of ACPs by RCEM [17] was considered by many to have contributed to the acceptability of employing NMPs in EDs/UTCs.

Some participants also reported that senior clinical and managerial decision makers familiar with NMPs and viewing them as of value, took that knowledge with them to other organisations and influenced the spread and adoption.

“..*a lot of it is probably to do with whether there's someone in senior management wanting to champion that* [NMPs] *as a kind of way of improving the workforce. ……..someone who's moved from somewhere else where they've been doing that, and then there’s kind of like a snowball effect I think sometimes….. they take that idea with them”*. Participant 11, national, lay.

Some participants considered that factors that were aiding the implementation of NMPs in urgent and emergency care came from all levels of the health system: the national policy statements by HEE and NHS England as well regionally funded training programmes. Several participants pointed to the value of training programmes that made explicit the level of NMP competency in emergency medicine to other clinicians, Examples were given of bespoke regional programmes as well as the credentialing process of RCEM [17]. The lay participants reported that, in the main, patients were content to be treated by the most suitable staff in terms of skills, knowledge, and experience rather than role. They reported the patient perspective as emphasising competent health professionals with good communication skills that engendered trust and confidence in the triage decisions.

*“You'll get very little about, 'It would have been better if I'd seen a doctor quick.' I don't think you will. I think people are much more…they want to just get to the right person who they can talk to and get treated.”* Participant 5, national, layperson

There were, however, also views expressed that a minority of patients expected to consult doctors and that there was considerable public confusion about who and what these new NMP roles were.

Participants described supportive factors for NMP employment as NMPs bringing a breadth of skills to the effectiveness of the team. Examples were given of NMPs from nursing and PA backgrounds bringing strong communication skills, NMPs from physiotherapist backgrounds having expertise in musculoskeletal conditions, NMPs from paramedic backgrounds having expertise in sudden deterioration of patient conditions and NMPs from pharmacy backgrounds having expertise in medicines.

“*I think the broadening of the workforce (with NMPs), and one of the things that brought me into emergency medicine…. I appreciate and value multidisciplinary working and I think it brings a far more holistic care to patients*. Participant 20, national, regional, clinician

Two participants commented that while there was some research evidence as to the contribution of NMPs in EDs/UTCs this was very limited and there was a need for more. This was viewed by some as one of the factors inhibiting the wider employment of NMPs, which we now report.

#### Factors perceived to be negatively influencing the implementation and future intentions for non-medical practitioners in the urgent and emergency care workforce

The reported hindering factors grouped into overlapping themes about: the extent of evidence of value of NMPs, the variation in scope of NMP practice and regulation with concomitant concerns about clinical risk; variation in workforce models deploying NMPs, the absence of long-term workforce plans with associated business plans, system inertia and professional resistance.

Some participants argued that there was very limited evidence as to the value, safety and benefit of NMPs in EDs/UTCs. Others considered that the gap was in knowledge transfer about value and safety to local organisations in a way that was useful to decision making:

*“I think a really clear articulation at a national level of actually what are the benefits, what are the risks? Where has this been done well? What can we expect from this workforce that might be different? So that can be something that's used for your local organisation.”* Participant 17, subregional, clinician

As reported earlier, commissioners were thought to be focused on out of hospital services to address problems in EDs/UTCs and some suggested that NMPs working outside of hospitals could help provide solutions rather than NMPs within EDs:

*“Because of the development of advanced practitioners, physicians’ associates, specialists in advanced practice, what we [ commissioners of services] can do is give people more treatment in their place of living.”* Participant 18, national, clinician, commissioner

New models of emergency department care were described, which were addressing the wider problems of patient flow out of EDs/UTCs, and which did not necessarily require NMPs in the ED. Examples were given of in-reach into the ED from consultant speciality teams, for example from geriatrics, that included speciality specific ACPs. Others suggested that there was increased use of other professional groups in the ED/UTC, such as paramedics and pharmacists, but this was not necessarily in NMP roles:

*“Some trusts are looking? at taking paramedics on to do front door streaming* [of the ED]*, not necessarily in that ACP role.”* Participant 13, regional, clinician, commissioner

Many participants pointed to the negative impact of the lack of national, regional and often local level health care workforce plans. The lack of funding was thought to deter the creation of training programmes; training posts with associated training and supervisor time; as well as NMP posts with associated medical supervisory time. A few participants argued that local detailed ED workforce planning based on the organisation context and patient demand should be used to inform regional and national workforce plans, rather than some national superimposed template of staffing. However, these participants were in unison in observing that there was an absence of long-term workforce planning.

*“The NHS only seems to really plan for the next 12 months from April to April, and we really struggle to persuade organisations that, actually, this is part of a five-year, ten-year plan, and they've got to invest now [in ACP in ED training] in order to have that positive effect much later on down the line”.* Participant 1, Regional, clinician, manager

A few participants described public sector organisations as incapable of swift, radical changes and consequently this was deterring wider NMP implementation. Variation between local level NHS organisations was cited as evidence of different attitudes to innovation. Devolved decision making within organisations was thought by some participants to result in risk averse choices rather than innovation.

*“I think there's real desire, actually, at the top of the trust to do this and to support this* [introduction of NMPs]. *But the long and short is the budget doesn't sit with the board. The budget sits with the operational units…..We're talking about them taking a risk. We're talking about them converting one type of post into another type of post. Or, actually even worse for them, investing to save…... But I think we're talking about such a fundamental shift that it becomes really hard for risk-averse divisional managers to, to decide to take that risk.*” Participant 17, sub-regional, clinician

The variation in scope of practice and training of different NMPs (i.e., leading to uncertainty in the competency and capability of NMPs), was identified by several participants as a factor that deterred inclusion of NMPs in ED/UTC workforces. This factor was mitigated, as described previously, for some participants by programmes that provided quality assurance of knowledge and skills; for example the RCEM ED-ACP programme [17]. A few participants pointed to a tension amongst senior decision makers as to how to standardise and quality assure competency amongst NMPs in EDs/UTCs.

*“At the moment, there is a want* [to fill medical vacancies]*, and there are those who would put a white coat on a clapping monkey and call it a clinician. You have to say, 'No, this has to be a quality assured measurement and training, because at the end of this is the patient's safety.”* Participant 20, national, regional, clinician

Individual professional organisations were thought to be ambivalent as to NMP roles and credentialing processes by other professional organisations. This was another factor that was considered to inhibit NMP development:

*“The organisations who represent the professions are more hesitant. The organisations who represent trust leaders or employers are much more pro, because we can see the value”*. Participant 6, national, policy

The absence of licensure by health professional regulatory bodies in the UK for advanced practice and for PAs, combined with linked restrictions (such as paramedics unable to prescribe certain classes of medications), was described as an ongoing deterrent for employers.

“*The lack of regulation over physician associates …..It's a real shame, because we could really benefit from seeing, for instance, prescribing rights. And that can only come with regulation. And it will just help in the workforce planning piece to know how far people can work up in terms of skill sets*”. Participant 6, national, policy

Finally, resistance within the medical profession to the development of NMPs roles was discussed by some participants. Only one participant reported direct objection from concern about future jobs:

*“I know there’s been a little bit of resistance when the ACP programme was first set up, from people who felt like, perhaps medics, who felt like their jobs might be, not be protected if they start training other people who can do quite a lot of their job”.* Participant 9, national, policy, commissioner

Medical consultants were described as considering a doctor as the ‘*gold standard*’ (Participant 17, sub-regional, clinician) in the workforce and consequently considered any other professional in that post would constitute a loss. Other participants reported more mixed views amongst doctors as to the acceptability or otherwise of NMPs. It was also pointed out that other professional groups could display negative behaviours towards the concept of NMPs.

*“They* [nurse ACPs] *also got kickback. They can get sometimes bullying, effectively from… Or snide remarks from their colleagues. 'Oh well, you're not one of us anymore because you're on that* [ACP rota]*.*” Participant 16, national, clinician

The tension between occupational groups was described by several participants as most frequently observed in the defence of staffing budgets against conversion to NMP posts. Staffing budgets were reported to be ringfenced to specific professional groups rather than the service in total.

*“For me, again, one of the big tensions is around developing these roles, 'Oh, well, where's the money coming from? Is it out of the nursing budget or the medical budget?”.* Participant 20, national, regional, clinician.

The ambiguity over the position of NMPs vis-a-vis both medicine and nursing, was considered to influence discipline specific budget holders not to support the use of their professions’ staffing finance for NMPs.

## Discussion

This study explored the macro and meso level factors influencing the implementation of NMPs in EDs/UTCs in England in the context of a National Health Service. While there are many studies of the introduction of NPs in primary care [41–43], to our knowledge this is the first study to explore this issue specific to EDs/UTCs from a national perspective, and the first for multiple types of professions in NMP roles. We found new evidence that suggested the numbers of NMPs were relatively small, unevenly distributed and faced uncertain growth. Similar views have been reported for multi-professional ACPs in all types of secondary care services in England [44].

In this first policy analysis study, we identified that the macro level policy in support of NMPs in EDs/UTCs was written in general terms rather than a prescription to the rest of the health service. Greenhalgh et al.’s model of implementation of innovation notes the importance of policy mandates in adoption of innovation. [33] The national policy statements on NMPs in EDs/UTCs had broadly desired implementation and outcomes i.e. they were normative in nature [45]. It has been argued that normative policies are subject to political contexts of ambiguity and conflict in implementation decisions [45]. Our finding that the employment of NMPs in EDs/UTCs was largely absent from the meso-level policies of NHS planning and commissioning organisations may be in part explained by the lack of directive from the national policy. Views of the national and regional level participants give weight to this explanation. The participants pointed to the absence of a national funded NHS England workforce plan as an inhibiting influence on further meso level actions in support of the spread and adoption of NMPs in EDs/UTCs. The absence of a national health care workforce plan has been commented on before in the NHS England context, although not specifically in the context of ED/UTC services [46]. A recent national recovery plan to improve ED/UTC patient waiting times and experience has committed (amongst other actions) to increasing the workforce throughout the urgent and emergency care system, including “increasing the numbers of advanced practitioners in priority areas, including emergency care “ (p 20, [47]). As part of this NHS England has required the local ICS to develop specific workforce plans for urgent and emergency care services [47]. On 1st June 2023 a new national NHS England workforce plan was published [48]. This committed to funding increased numbers of advanced practice and physician associate training places, with specifically 150 advanced practice paramedic training places a year to support the same day emergency care initiative. The national plan committed to work with local level ICS organisations to implement the policy, however, it remains to be seen if the implementation plans are more prescriptive. Further longitudinal study is required on the impact of these national and sub-regional workforce plans on the numbers and spread of NMPs in ED/UTCs in England.

Participants identified that areas with long standing, and acute shortages of emergency medicine doctors were more receptive to the innovation of NMPs in their workforce. In Greenhalgh et al.’s model this represents readiness for change of an unworkable situation [33]. The COVID-19 pandemic experience of having to innovate suddenly in workforce deployment and ways of working was considered by participants to have also contributed to system and service readiness for change. Participants reported that evidence of value and acceptability of NMPs to services, clinicians and patients was influential in supporting the spread and growth of NMPs; that is NMPs demonstrated the relative advantage as described in Greenhalgh et al.’s model for adoption of innovation [33]. However, there was also uncertainty and differing views amongst participants. Divergent views were offered as to whether there was publicly available evidence of clinical safety, efficiency, and cost effectiveness i.e., relative advantage of having NMPs in EDs/UTCs. Synthesis of evidence, primary research and modelling over long-term periods is required to provide clinicians and managers with the information to decide on the relative advantage, or otherwise, of NMPs.

There were also differing views as to which of the following problems NMPs were part of the solution to: medical shortages, problematic patient flow into and through EDs, failure to retain experienced ED nursing staff or lack of staffing continuity resulting from training rotations of doctors. Further ambiguity was identified as to which workforce models involving NMPs should be pursued to improve ED/UTC experience and services. Participants offered the following variety: NMPs in primary care to reduce patient demand at EDs/UTCs, NMPs attached to secondary care specialties to enhance in-reach into EDs/UTCs for specific groups of patients, NMPs with prior professional backgrounds to target specific patient groups in the ED/UTC e.g., physiotherapists and patients with musculoskeletal problems and NMPs working generically within the ED/UTC as senior clinical decision makers to help improve patient flow overall. Greenhalgh et al.’s model proposed that for successful introduction the innovation must be understood by all parties and be compatible with current ways of working [33]. Apart from differing NMP workforce models, participants reported uncertainties derived from varied NMP training, competencies, regulation and governance, as well as confusion among patients and the public. Counterbalancing these uncertainties were the macro and meso level work by some organisations to provide benchmarked training and accreditation for some types of NMPs working in ED/UTCs [17]. Further study over time is required to investigate whether these developments impact on decisions to include NMPs or not in ED/UTC workforces.

In the absence of national or regional policies, participants pointed to the positive impact of change management activities by individuals at the meso and micro-organisation level such as senior influential champions, which features in Greenhalgh et al.’s model [33] and has been described many times in relation to the introduction of NP’s roles in individual organisations [49, 50]and in ED/UTC settings [21, 22].

Multiple reviews of the introduction and spread of advanced and expanded health profession roles have pointed to national health workforce policies as enabling but that these have been dependent on the extent of opposition or support of other professions, most notably medicine [51–54]. Our documentary policy review provided evidence of active support at the national level by medical organisations to NMPs in ED/UTC, although the interviews provided a more nuanced view at the meso organisational level. Participants reported mixed views amongst doctors, with some being actively supportive, some that were neutral and some being unsupportive for a variety of reasons. These reasons included the erosion of medical roles and medical training opportunities, uncertainty as to the capability of NMPs and concern about the adequacy of the funding for medical supervision for NMP roles. Surveys of emergency medicine doctors in Australia and the US have similarly reported divided opinions [55, 56]. The innovation of NMP roles is fundamentally a disruption to current ways of working and the work boundaries between professions. Abbott’s seminal work offered a theory of the system of professions, characterized as a jostling, interdependent ecology in which the activities of one occupational group impact on others and are tied up with issues of power, status, and rewards [57]. Abbott argued that at the micro level of the team, these boundaries were always blurred or fuzzy, and it was at the organization and broader societal level that the boundaries were significant in claims for professional jurisdiction, clients, knowledge, resources and rewards [57]. The uncertainties on regulation, governance and multiple credentialling systems, which were reported by participants as inhibiting factors to the development of NMPs in EDs/UTCs, could be viewed as evidence of the national level jostling between some professional organisations for jurisdiction and resources. The reported preference not to redeploy resources from one professional group (i.e., medicine or nursing) to long term funding of NMP posts in EDs/UTCs will continue to be inhibiting to wider spread and adoption.

Further investigation is required to consider whether these findings are generalisable to other specialties, and to similar health systems in other countries.

### Limitations and strengths

We identified three limitations of the study. The first limitation was that the mixed methods data collection was dependant on publicly available documents of NHS organisations and details of senior NHS postholders, which proved less accessible at the meso level due to NHS reorganisations before and during the study. We tried to mitigate this through a systematic method of national and regional website searching and cross referencing. A second limitation was the smaller number of senior participants interviewed than we had planned, in part due to the timing of approaches through surges of the COVID-19 pandemic and associated public health campaigns. We were able to ensure that participants who were able to be interviewed represented diversity in levels, regions and organisations of the health system. The third limitation was that some of the research team have investigated new roles in the NHS through a number of funded studies which may have influenced their interpretation of the findings from prior knowledge, although this could also be a strength. Input from the wider team helped mitigate this as it included members who have not undertaken investigations of this topic and brought diverse clinical, managerial, and academic discipline perspectives. A strength of this study use of mixed methods which enabled data collection at both macro and meso levels. A further strength was the study was framed theoretically, and this was used in the analysis and to propose new lines of empirical enquiry.

## Conclusion

This study investigated the implementation of NMPs in EDs/UTCs in England from macro and meso perspectives synthesizing evidence from documentary analysis and interviews. The evidence suggested the numbers of NMPs were relatively small, unevenly distributed and growth uncertain. A broad macro level policy support was identified but in a general rather than prescriptive form which appeared to result in little tangible support at the meso level of the system. Subsequent to the study, further national policy has been issued directed to the recovery of the urgent and emergency services and increased employment of NMPs [47]. Longitudinal study is required to identify the extent to which this is implemented and whether factors identified in this study endure. While we identified many features of a system ready to introduce workforce innovation there appeared to be much ambiguity surrounding NMPs in EDs/UTCs and the potential for conflict with other professional groups. There were many uncertainties reported about regulation, governance and multiple credentialling systems of NMPs in ED/UTCs. This warrants consideration over time too and in the context of other health systems where regulation has been established. An additional reported area of ambiguity was whether or not there was research evidence documenting the relative advantage of including NMPs within the staffing of ED/UTCs. The production of such evidence, disseminated in a format easily accessible to senior clinicians and managerial policy makers requires urgent attention for short- and long-term workforce planning. Further investigation is required to consider whether these findings are generalisable to other specialties, and to similar health systems in other countries.

### Supplementary Information

**Additional file 1.**

## Data Availability

The datasets used and/or analysed during the current study are available from Dr Mary Halter on reasonable request.

## Abbreviations

ACP: Advanced Clinical Practice
ED-ACP: Emergency care—advanced clinical practice
ED/UTCs: Emergency departments and urgent care treatment centres
FTE: Full time equivalent
HEE: Health Education England
NHS: National Health Service
NMP: Non-medical practitioner
NPs: Nurse practitioners
PAs: Physician associates/assistants
RCEM: Royal College of Emergency Medicine
STPs/ICS: Sustainability and Transformation Partnerships and Integrated Care Systems
UK: United Kingdom
USA: United States
WHO: World Health Organisation
