# Supplementary material for: Non-medical practitioners in the staffing of emergency departments and urgent treatment centres in England: a mixed qualitative methods study of policy implementation
Source: BMC Health Serv Res. 2023 Nov 8;23:1221. doi: 10.1186/s12913-023-10220-4 (PMC10631061; doi:10.1186/s12913-023-10220-4)
Supplement: Supplementary file 1 — Additional file 1. [file 12913_2023_10220_MOESM1_ESM.docx]

**Implementation of the non-medical practitioner workforce into the emergency and urgent care system skill-mix in England: a mixed methods study of configurations and impact**

**
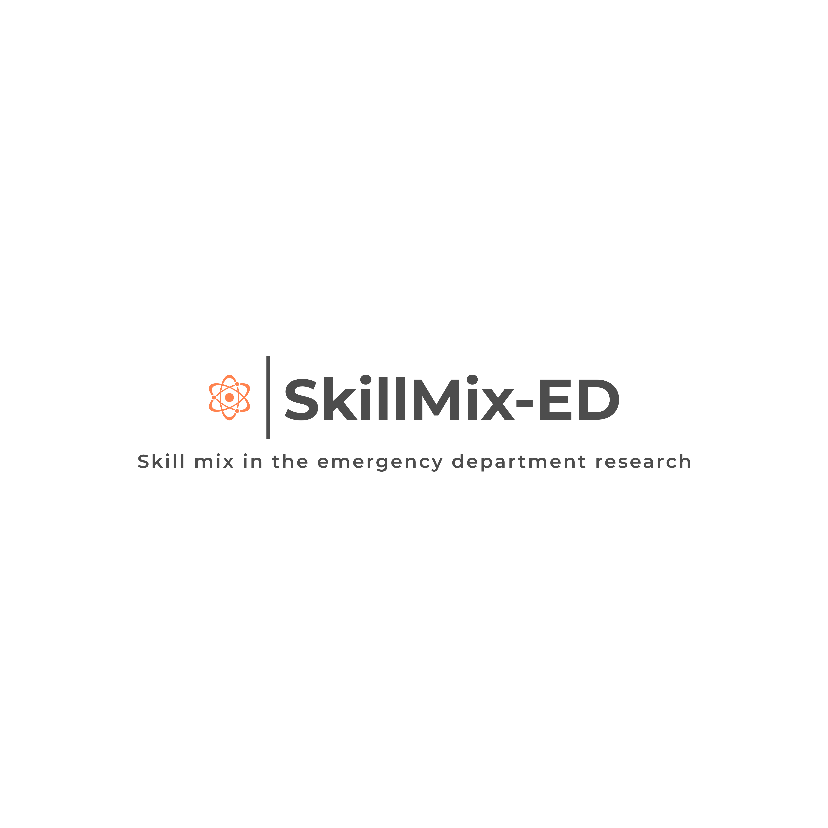
**

**TOPIC GUIDE**

# Interview with senior clinicians, commissioners, managers and lay representatives.

## Instructions for researcher

Confirm that the interviewee understands:

- the purpose of the research,
- what the interview entails,
- how confidentiality and anonymity will be assured,
- that they have had chance to ask questions,
- they are content to be recorded digitally or not,
- they can stop at any time without explanation.

Confirm the consent form is signed/returned.

Confirm with the participant at the start of the digital recording that they have consented to the recording.

## Topic areas for open questions

1. Participant’s exposure to non-medical practitioners (NMPs) in ED/UTC

- Confirmation of the participant’s role/post/job and involvement in emergency /urgent care
- Ask them to describe any experience with NMPs in emergency services (check the range e.g., nurse practitioners, physician associates, paramedics, others e.g., pharmacists)
  - Probe if any aspect unclear e.g., over what time period, type of involvement e.g., working alongside or in commissioning services only

1. Participant’s experience of influences/rationale in decisions to employ/train NMPs

- Can you describe the factors influencing how your [add organisation] views decisions regarding training/employing?
  - Probe on different types of factors from the literature e.g., medical workforce shortages, increasing patient demand, concerns about patient experience and outcomes
- If appropriate to their role – ask them to describe the expectations as to how NMPs were/are expected to impact on patient experience and outcomes and staff experience and outcomes.

1. Participant’s experience as to impact or otherwise of NMPs in emergency departments (EDs)/urgent treatment centres (UTCs)
   - Explore experience of any positive or negative experience – what type, on who, from who/which perspective, on what aspect of the service delivery?
   - Knowledge of any unintended consequences of NMPs in ED/UTC or subsequent changes in deployment/training /utilisations?
   - Probe for any unpublished or published evidence of impact – particularly regarding patients’ views – obtain details /source.
2. Participant’s experience of growth or otherwise of NMPs in ED/UTC
   - To what extent to your knowledge have these roles grown or spread or become embedded in [add domain of knowledge region/organisation]?
   - What factors have supported or inhibited the development /growth or embeddedness of NMPs?
     - Probe on types of people/professions/commissioners/resources/change management techniques /issues for both types of factors
3. Participant’s experience /views as to the right balance of skill mix in EDs and UTCs
   - Any guidance/rules of thumbs /mechanisms for deciding this?
   - Any views on judging whether skill-mix right? Or needs adjusting? Any particular criteria which would be indicative?
   - Any views as to what guidance on skill-mix would be beneficial to clinical /managerial/commissioning staff?
4. Any other views or points? Or anything to add? Any others the research team should contact who may have undertaken more work/engaged with this question?

**Thank the participant.**

**Confirm process of permissions for retention of contact details for publications and information about the study.**
